# Outcome of patients with acute bacterial meningitis in a teaching hospital in Ethiopia: A prospective study

**DOI:** 10.1371/journal.pone.0200067

**Published:** 2018-07-18

**Authors:** Esayas Kebede Gudina, Markos Tesfaye, Andreas Wieser, Hans-Walter Pfister, Matthias Klein

**Affiliations:** 1 Department of Internal Medicine, Jimma University, Jimma, Ethiopia; 2 Centre for International Health, Ludwig-Maximilians-University, Munich, Germany; 3 Department of Psychiatry, St. Paul’s Hospital Millennium Medical College, Addis Ababa, Ethiopia; 4 Department of Bacteriology, Max von Pettenkofer Institute, Ludwig-Maximilians-University, Munich, Germany; 5 Division of Infectious Diseases and Tropical Medicine, Medical Center of the University of Munich, Ludwig-Maximilians-University, Munich, Germany; 6 German Center for Infection Research (DZIF), Partner Site Munich, Munich, Germany; 7 Department of Neurology, Klinikum Grosshadern, Ludwig-Maximilians-University, Munich, Germany; Aga Khan University - Kenya, KENYA

## Abstract

**Background:**

The mortality and neurologic sequelae associated with acute bacterial meningitis (ABM) remain high despite advances in medical care. The main aim of this study was to evaluate short-term outcome in patients treated as bacterial meningitis at a teaching hospital in Ethiopia to identify factors that could be focused on to improve outcome in this setting.

**Methods:**

A hospital based longitudinal study was conducted at Jimma University Hospital in southwest Ethiopia from March 1, 2013 to December 31, 2015. Participants of this study were patients of age 18 years and older who were treated as confirmed or possible cases of ABM. Patients were followed throughout their hospital stay for change in their clinical course and predefined end points. A multivariable analysis was done to identify factors associated with unfavorable outcomes.

**Result:**

90 patients admitted with diagnosis of acute bacterial meningitis were included in the study; cerebrospinal fluid was analysed for 85 (94.4%) of them. Causative bacteria were isolated in 26 (28.9%) patients only; most of these isolates (84.6%) were either *Streptococcus pneumoniae* or *Neisseria meningitidis*. Patients managed as cases of ABM at the hospital suffered from a high rate of unfavorable outcome (36.7%) and an overall mortality rate of 22.2%. Impaired level of consciousness (AOR = 0.766, 95% CI = 0.589–0.995), dexamethasone therapy (AOR = 4.676, 95% CI = 1.12–19.50) and fever persisting after two days of admission (AOR = 24.226, 95% CI = 5.24–111.96) were found to be independently associated with unfavorable outcome.

**Conclusion:**

Outcome in patients treated for ABM at the hospital was found to be poor. Impaired mentation, treatment with adjunctive dexamethasone and persistent fever were found to be associated with poor outcome. Thus, development of clinical guidelines for treatment of ABM that suit the local context is essential to improve patient management and outcome.

## Introduction

Acute bacterial meningitis (ABM) is among the ten most common infectious causes of death [1] and is responsible for approximately 135,000 deaths annually throughout the world [2, 3]. It remains a devastating disease despite advances in medical care [4–6]. The case fatality rates differ among various studies and reach 6–17% in pneumococcal meningitis in high income countries [7, 8]. Moreover, half of the survivors may have some form of neurological sequelae [5, 9]. In resource-poor settings, particularly in areas with high HIV prevalence such as sub-Saharan Africa, the mortality can be as high as 50% [10–12]. Delayed presentation and treatment initiation [13], limited diagnostic facility and poor standards of care [14] are some of the major reasons for poor outcome of meningitis in Africa. For these reasons, management of patients with suspected meningitis presents an exceptional challenge to physicians working in resource-poor settings [14].

The occurrence of HIV infection has dramatically changed the spectrum of central nervous system (CNS) diseases in sub-Saharan Africa [15]. While *Streptococcus pneumoniae* and *Neisseria meningitidis* remain important causes of adult meningitis in immunocompetent patients [10, 16], cryptococcal meningitis (CCM) and tuberculous meningitis (TBM) have now become the commonest causes of meningitis in HIV infected patients in Africa [17–19]. An important challenge of these two causes of meningitis is their misdiagnosis for bacterial meningitis [20].

An accurate and confirmatory microbiological diagnosis of meningitis is difficult in African hospitals. Consequently, management of suspected meningitis is mainly clinical and prompt treatment remains a challenge [14]. Due to repeated outbreak of meningococcal meningitis in the meningitis belt of Africa [21, 22], most patients with suspected meningitis are often started on treatment for pyogenic meningitis without due consideration for differential diagnoses. As a result, diagnosis of other causes of meningitis such as TBM is mainly clinical and often after a failed antibiotic trial for ABM [17, 23, 24].

In addition to these facts, dexamethasone, despite lack of evidences, is still recommended as adjunctive therapy in the management of suspected bacterial meningitis in certain parts of Africa [25, 26]. This, besides absence of benefit, may also contribute to poor outcome of patients with CCM [27] and TBM who are not otherwise started on proper antimicrobial therapy [28]. Because of all these challenges in their diagnosis and management, both CCM and TBM are associated with very high case fatality in Africa [23, 29, 30].

Ethiopia is one of the countries in the African meningitis belt [31]. Most documented data regarding epidemiology of meningitis in adults in Ethiopia are limited to surveillance and retrospective outbreak reports for meningococcal meningitis [32–35]. As a result, information regarding sporadic bacterial meningitis in the country is scarce. Moreover, the overall incidences and spectrum of complications, and prognostic factors in adults with meningitis in Ethiopia are not well known. In addition to these, the underdeveloped healthcare system has made care of patients with meningitis challenging. Most important, according to retrospective data, the diagnosis is based mainly on clinical presentations and treatment is entirely pragmatic [36].

In this study, we aimed to prospectively assess the discharge outcome of patients treated as acute bacterial meningitis and factors associated with unfavorable outcome in a tertiary care teaching hospital in Ethiopia to identify factors that could be focused on to improve outcome in this setting.

## Research design, methods and procedures

### Settings

This study was conducted at Jimma University Hospital, a public teaching hospital located in Jimma town in southwest Ethiopia. It is the only tertiary care centre in southwest Ethiopia that has a catchment population of over 15 million.

#### Study design and duration

This is a hospital based longitudinal observational study of all patients treated at the hospital with ABM as the most likely diagnosis. Time period of the study was from March 1^st^, 2013 to December 31^st^, 2015 (34 months). Patients who were admitted with diagnosis of ABM at the hospital during the study period were prospectively followed throughout their hospital stay.

### Participants

Participants of the study were adults of age 18 years or older at the time of hospitalization who were treated as confirmed or possible cases of ABM. For this study, the patients were grouped into three categories based on clinical and laboratory evidences for the diagnosis of ABM.

1. *Confirmed bacterial meningitis*–when the diagnosis of bacterial meningitis was confirmed by detection of causative bacteria using culture, Gram stain microscopy or latex agglutination test (LAT) from cerebrospinal fluid (CSF) specimen.
2. *Bacterial meningitis with unidentified etiology (pyogenic meningitis)*–for patient who had at least three of the following CSF findings: (1) Turbid CSF, (2) >1000 leucocytes/μL, (3) Protein >100 mg/dl and (4) CSF to serum glucose ratio of <0.4 AND an undetected pathogen
3. *Possible bacterial meningitis*–(**1**) for those who had lumbar puncture (LP), abnormal CSF but not fulfilling the above criteria: (>100 leucocytes/μL but <1000 OR >10 PLUS glucose ratio<0.4 PLUS protein>100 mg/dL) AND no evidence for alternative diagnosis for these changes. (**2**) In cases where LP was not possible, presence of all triads of bacterial meningitis for no more than 7 days: (i) Fever> 39°C, (ii) nuchal rigidity (iii) mental status change PLUS negative blood film for *Plasmodium* species PLUS absence of clinical signs and symptoms for other differential diagnoses

### Exclusions

Meningitis suspected cases that were treated as other forms of meningitis (other than ABM) from the outset and those with posttraumatic meningitis were automatically excluded from the study. Patients initially started on antibiotics as suspected cases of ABM but whose differential diagnosis of bacterial meningitis was dropped by the treating physicians after the arrival of CSF results or based on other clinical parameters were left out of the study as well. Patients who completed treatment for possible bacterial meningitis but did not have supportive laboratory or clinical evidences were also excluded from this report.

### Selection of study participants

All patients who fulfilled the inclusion criteria and willing to participate on the study were recruited consecutively.

### Data collection procedures

The data was collected using a pretested structured questionnaire. The patients or the guardian (s), if the patient was unconscious, were asked for consent before data collection. Then, an interview was performed to obtain information regarding socio-demographic profiles, duration of illness and symptoms at presentation. Lumbar puncture, in the absence of contraindications, was performed under aseptic conditions for all patients with suspected ABM to collect CSF specimen. This was done as soon as it was possible or within 24 hours of hospital presentation. CSF specimen was collected in two separate sterile tubes (2-3ml each) to perform the following tests:

1. Physical analysis (gross appearance), biochemical test (glucose and protein) and microscopy (leucocyte with differentials and Gram stain).
2. Culture and antibiotic susceptibility test, and antigen test (LAT) for common bacteria causing meningitis. About 1-2ml of specimen from the second tube was saved in -20°C freezer for possible further molecular analysis.

LAT was done using the bacterial antigen kit (Wellcogen^**®**^ Bacterial antigen kit, UK) designed to detect 5 groups of bacteria: Group B *Streptococcus* (GBS), *Haemophillus influenzae* type B (Hib), *Streptococcus pneumoniae*, *Neisseria meningitidis* ACY W and *Neisseria meningitidis* B/*Escherichia coli* K1.

Antibacterial susceptibility was determined in vitro by Kirby-Bauer disk diffusion method following European Committee on Antimicrobial Susceptibility Testing (EUCAST) guidelines.

Acid-fast bacilli staining and microscopic evaluation of the specimen was also performed for all patients who had a LP. In all HIV patients, Indian ink staining of the specimen was done and the specimen was inoculated onto Sabouraud dextrose agar for detection of fungal pathogens. The reports of laboratory findings were documented on a report format specifically prepared for this study.

Both biochemical tests and microbiological analysis from CSF and other laboratory tests were performed at the hospital as part of routine diagnostic services. However, latex agglutination test (LAT) which was not routinely available in public hospital in Ethiopia was done only as part of this research project. Blood culture, cryptococcal antigen test and PCR services were not part of routine hospital service during the study period and were not done. Public diagnostic laboratories in Ethiopia are supported and accredited by the Ethiopian Public Health Institute.

### Inpatient follow-up and outcome assessment

Patients were evaluated daily for symptom improvement or occurrence of new symptoms. Vital signs were assessed every four hours for the first 48 hours and based on the need thereafter. Daily follow-up with a neurosign chart that included the following variables—Glasgow coma scale (GCS), seizure, headache, and nuchal rigidity was done during the inpatient treatment. Occurrence of death was documented along with the days stayed in the hospital. Discharge outcome was assessed using Glasgow outcome scale (GOS) [37]. Patients were also assessed at discharge for gross neurologic deficits (visual problems, hearing deficit, and body weakness) and mini-mental state examination.

HIV status was determined by rapid test within three days of admission for all patients. CD_4_ count and routine evaluation for status of HIV infection were performed before discharge when possible. Otherwise, patients in stable condition were discharged and given a short appointment for initiation of HIV treatment.

### Data quality control

All clinical evaluations were performed by clinicians who were experienced in care of patients with infectious diseases. The data was double entered to minimize errors.

### Data processing, analysis and interpretation

The data was de-identified, cleaned, edited, entered into the computer and analysed using SPSS (IBM SPSS Statistics for Macintosh, Version 20.0. Armonk, NY). Frequency and percentage were used to summarize categorical variables. Normally distributed continuous variables were presented as mean and standard deviation and skewed data were presented as median with interquartile range. Chi-square test was performed to assess the binary association between various categorical variables. For normally distributed continuous variables, Student’s t-test for independent samples was used. Non-parametric (Mann-Whitney-U) test was used to compare skewed continuous variables (CSF white cell count, CSF protein and glucose ratio). Bivariate analysis was done to identify association between dependent and independent variables. All independent variables with p<0.05 in bivariate analysis were entered for multivariable analysis. Forward logistic regression analysis was done to identify the best fit model. Independent predictors were analysed for Glasgow outcome scale on discharge. P-value of < 0.05 was used as level of statistical significance.

Complete demographic and clinical data were available for all patients. However, five patients who did not have lumbar puncture had missing CSF results. In such instances, cases with missing data were excluded from multivariable analysis.

### Outcome measurements

The primary outcome measurements were death and Glasgow outcome scale on leaving the hospital. The overall discharge outcome was dichotomized into favorable outcome (GOS = 5) and unfavorable outcome (GOS = 1–4) [37]. Neurologic weakness at discharge, and length of hospital stay were other outcome measurements.

### Ethical considerations

The study was approved by institutional ethical review board of College of Health Sciences at Jimma University (reference letter RPGC/24/2013). Written informed consent was obtained from the participants (the guardian if the patient was unconscious at presentation). Only patients’ initials and medical registration numbers were used for data collection. The collected data was coded and de-identified before it was entered to computer for analysis.

## Results

### Study participants (Baseline characteristics)

**Fig 1:**
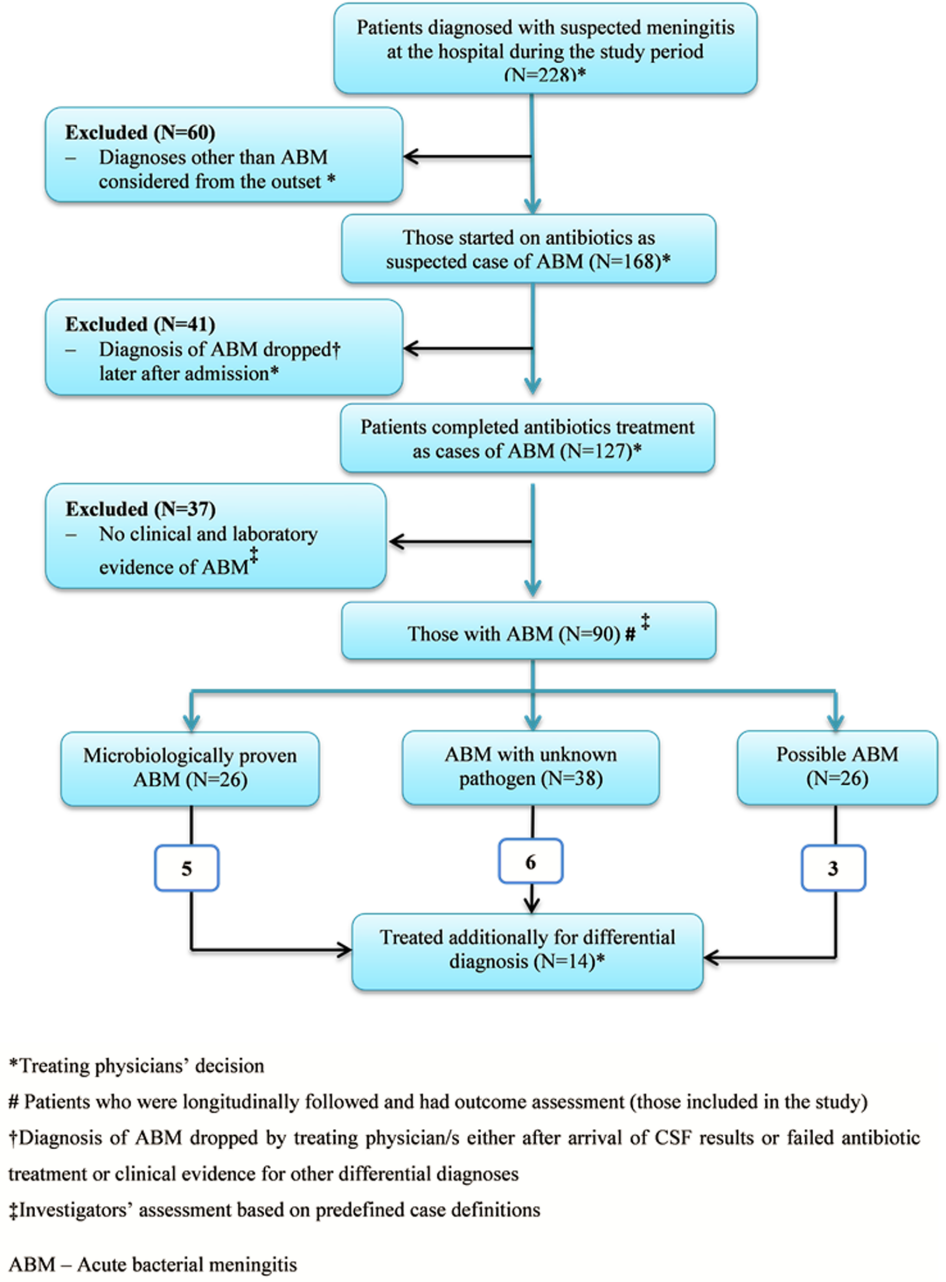
A CONSORT flow diagram for patients treated for suspected meningitis and those included in the study as cases of bacterial meningitis at Jimma University Hospital, Ethiopia.

A total of 228 patients were managed as cases of meningitis at the hospital during the study period of 34 months. Of these, 127 patients were treated as cases of bacterial meningitis. However, only 90 patients had clinical and/or laboratory evidences for bacterial meningitis and included in the study and prospectively followed. Among those included in the study, the diagnosis of ABM was proven by identification of a causative pathogen in 26 patients only. Another 38 patients fell into category of culture negative bacterial meningitis. In 26 patients, the diagnosis of bacterial meningitis remained possible but was not excluded. In 37 patients treated as bacterial meningitis, the diagnosis was not supported by clinical and laboratories evidences (Fig 1).

The mean age of the participants was 32.3 years (SD = 13.1); 85.6% of them were younger than 50 years. Male participants (58; 64.4%) and rural residents (58; 64.4%) constituted for the majority. The duration of illness before presentation was 4.5 days (SD = 3.6) (Table 1).

**Table 1:** Baseline characteristics of patients treated as acute bacterial meningitis at Jimma University Hospital, Ethiopia.

Characteristics	Total, (N = 90)	Proven ABM, (N = 26)	ABM with unknown etiology (N = 38)	Possible ABM (N = 26)
Age, Mean (SD)	32.3 (13.1)	31.4 (14.2)	32.7 (13.0)	32.8 (12.5)
Gender, N (%)				
Male	58 (64.4)	18 (69.2)	23 (60.5)	17 (65.4)
Female	32 (35.6)	8 (30.8)	15 (39.5)	9 (34.6)
Residence, N (%)				
Rural	58 (64.4)	16 (61.5)	24 (63.2)	18 (69.2)
Urban	32 (35.6)	10 (38.5)	14 (36.8)	8 (30.8)
Duration of illness in days, Mean (SD)	4.5 (3.6)	4.6 (4.1)	4.6 (3.6)	4.5 (3.3)
Symptoms/signs, N (%)				
Headache	88 (97.8)	25 (96.2)	38 (100)	25 (96.1)
Fever	87 (96.7)	26 (100)	35 (92.1)	26 (100)
Nuchal rigidity	76 (84.4)	20 (76.9)	35 (92.1)	21 (80.8)
Vomiting	70 (77.8)	20 (76.9)	30 (78.9)	20 (76.9)
Impaired consciousness	50 (55.6)	12 (46.2)	21 (55.3)	17 (65.4)
Photophobia	35 (38.9)	10 (38.5)	12 (31.6)	13 (50)
Seizure	20 (22.2)	3 (11.5)	6 (15.8)	11 (42.3)
Focal neurologic deficit	3 (3.3)	0	1	2
Hypotension	7 (7.8)	3	0	4
Pulmonary crepitation	19 (21.1)	7 (26.9)	7 (18.4)	5 (19.2)
Antibiotic treatment before presentation, N (%)	29 (32.2)	9 (34.6)	10 (26.3)	10 (38.5)
HIV infection, N (%)	16 (17.8)	7 (26.9)	6 (15.8)	3 (11.5)

ABM—acute bacterial meningitis

Nine (10%) patients reported regular use of alcohol and 7 (7.8%) were current smokers. Three (3.3%) of the participants were known diabetic patients and 12.9% (4/31) of reproductive age women were pregnant. Three (2.4%) patients had recent contact with patients of similar illness.

### CSF characteristics

Lumbar puncture to collect CSF specimen was performed in 85 (94.4%) of patients. In four patients, LP was deferred due to contraindications (deep coma with GCS of ≤5 or focal neurologic deficit or cranial nerve palsy). In the remaining one patient, the attempt to collect a CSF specimen failed due to technical reasons. Of those who had the CSF analysed, the specimen was collected after at least the first dose of antibiotics in 45 (52.9%) patients; 21 (24.7%) had taken antibiotics for at least 24 hours before LP.

**Fig 2:**
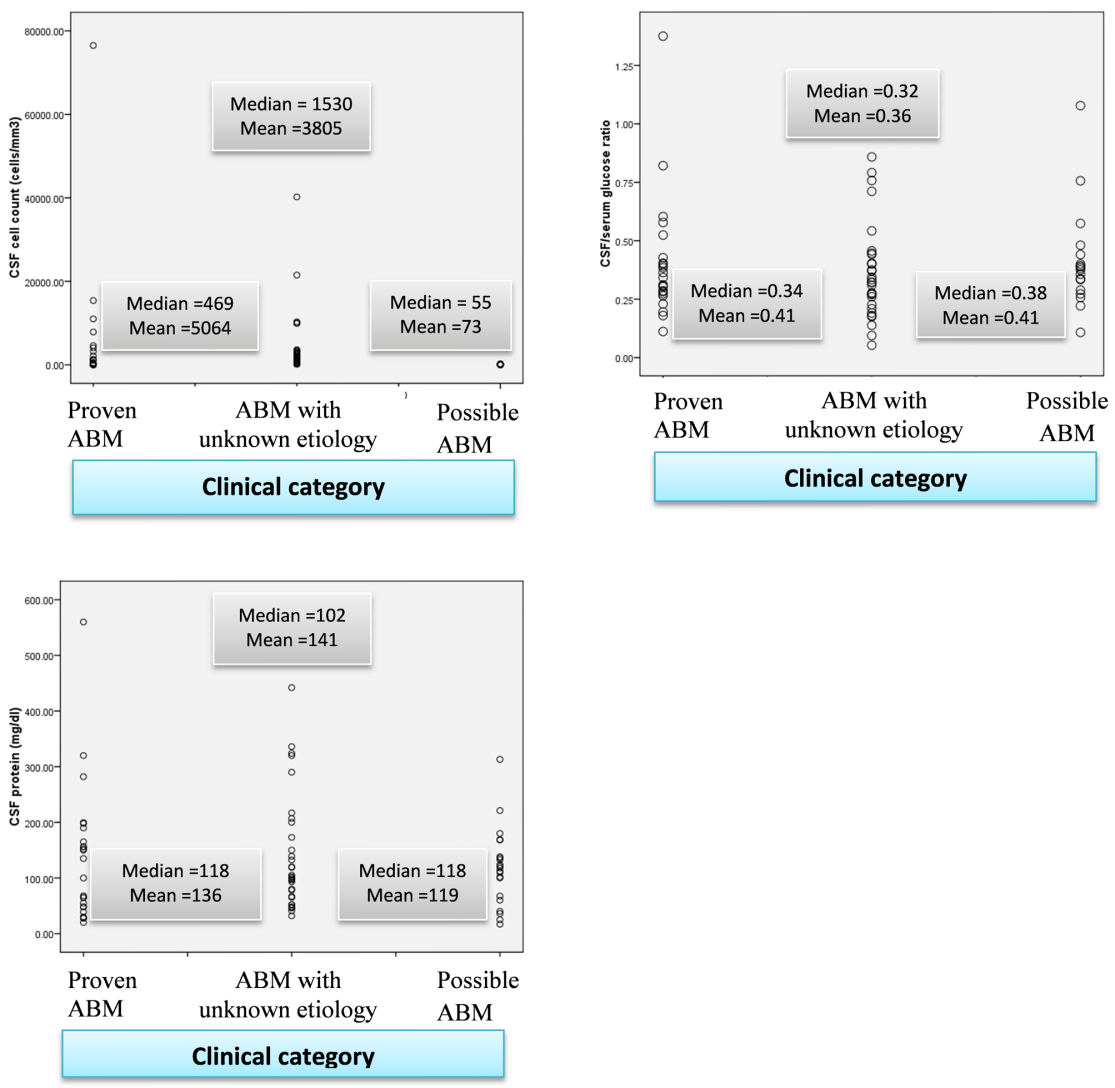
Scatterplot showing CSF profile of patients admitted with diagnosis of acute bacterial meningitis at Jimma University Hospital, Ethiopia.

In 56 (65.9%) of them, purulent CSF was collected of which 52 were visibly turbid. The median white cell count in the CSF was 700 cells/μL; 73% of the cases had CSF pleocytosis of >100 cell/ μL. The median CSF protein and glucose ratio were 110 mg/dL and 0.34 respectively. Patients with proven ABM and ABM with unidentified etiology had higher CSF cell count, higher CSF protein and lower glucose ratio as compared with possible ABM cases (Fig 2).

### Identification of etiologic agents

Among those who had their CSF analysed (85), causative bacteria were detected in 26 (30.6%) patients. *Streptococcus pneumoniae* was identified in 13 (50%) of them; nine (34.6%) were *Neisseria meningitidis* ACYW and three were *Haemophilus influenzae* type b cases. One patient, with advanced HIV infection and *Strongyloides stercoralis* hyper-infestation, had *Escherichia coli* meningitis.

Most of the proven cases, 23 (88.5%), were detected by Gram stain and 15 (57.7%) were positive for LAT. However, only 14 (51.9%) of those microbiologically confirmed cases were isolated on culture; eight cases were identified on all the three microbiological tests (Gram stain microscopy, LAT and culture). Nine of the proven ABM cases had been given at least one dose of oral or parenteral antibiotics before LP.

Eight of the culture isolated organisms were *Streptococcus pneumoniae* cases. With regard to antibiotic susceptibility pattern, all the eight culture positive *S*. *pneumoniae* cases were found to be susceptible to ceftriaxone and seven were susceptible to penicillin.

Only three of the *Neisseria meningitidis* ACYW cases were isolated from culture; all were susceptible to penicillin. One isolate was grown at a time when ceftriaxone e-tests were not available and showed a small inhibition zone around the ceftriaxone disc (<33mm for 30μg ceftriaxone disc). The isolate was frozen but could not be re-grown for final analysis, thus the ceftriaxone MIC of the last isolate remains unclear, reduced susceptibility was assumed for this isolate.

The *Escherichia coli* isolated in HIV patient showed an extended spectrum beta-lactamase phenotype, being resistant against penicillin, amoxicillin/clavulanic acid and third generation cephalosporin. It was found to be susceptible to gentamicin only among locally available antimicrobials.

### Other laboratory profiles

All patients had a complete blood count and peripheral blood film test for hemoparasite during their current admission. Sixty-one patients had analysis of erythrocyte sedimentation rate (ESR). The median white count, hemoglobin, platelet count and erythrocyte sedimentation rate (ESR) were 11,575 cells/μL (IQR = 9850), 12.3 g/dL (IQR = 3.3), 248,000 (IQR = 148,000) and 40 mm/hr (IQR = 46) respectively (Table 2). Peripheral leucocytosis (white count >11,000/μL) was detected in 50 (55.6%) patients; 40 (44.4%) had anemia as defined by World Health Organization (13 g/dL for men and <12 g/dL for women), but only nine (10%) had severe forms (hemoglobin <8 g/dL).

**Table 2:** Other laboratory profile of patients treated as acute bacterial meningitis at Jimma University Hospital, Ethiopia.

Laboratory findings	Total (N = 90)	Proven ABM (N = 26)	ABM with unknown etiology (N = 38)	Possible ABM (N = 26)
White cell count (cells/mm^3^), median (IQR)	11575 (9850)	15300 (14995)	12050 (9425)	10800 (8440)
Hemoglobin (g/dL) median (IQR)	12.3 (3.1)	12.4 (3.6)	12.7 (3.0)	12.2 (3.8)
Platelet (cells/mm^3^), median (IQR)	248000 (148000)	211000 (164500)	234000 (117000)	233000 (146000)
ESR (mm/hour), median (IQR)	40 (46)	25 (68.5)	40 (39)	46 (54)

ESR—erythrocyte sedimentation rate

Among HIV positive patients, eight of the 10 who had CD4 count recently had <200 cells/μL. On WHO’s clinical staging, all of the HIV patients were at stage 3 or 4.

### Case management

Ceftriaxone given as 2 gm intravenously twice daily was the main stay of treatment; used as the only antibiotic in 60 patients and in combination with other antibiotics in 29 patients. In one patient, a combination of crystalline penicillin and chloramphenicol was used. Overall, 30 (33.3%) patients were given adjunctive dexamethasone treatment.

Fourteen patients (five of microbiologically proven ABM, six from culture negative ABM, three from possible ABM) were additionally treated for other differential diagnoses (Fig 1). Twelve of them were treated as suspected cases of tuberculous meningitis even though the diagnosis was not confirmed. Treatment for alternative diagnosis in these patients was initiated after initial antibiotic trial as evidenced by a delay of 2.4 days (SD = 1.3) before treatment commencement for differential diagnoses.

### Hospital course and discharge outcome

Patients were followed daily with vital sign assessment and neuro-sign charts. Five patients died within the first 48 hours of hospital admission. Most of the survivors responded well to the first few days of antibiotic therapy. However, in 28 (32.9%) patients, fever persisted beyond the second day of inpatient antibiotic treatment. Among patients who had impaired consciousness on presentation, 26 (52%) regained consciousness within the first 3 days of inpatient treatment.

The average length of hospital stay was 13 days (SD = 9) with range of 12 hours to 43 days. There is no difference between the groups.

### Outcome on leaving hospital

The overall mortality was 22.2% (20 deaths). Overall, 33 (36.7%) patients had an unfavorable outcome (GOS<5) at discharge. Fifty-five (61.1%) patients were discharged with improvement, two left the hospital in the same condition like at the time of admission, and additional 13 (14.4%) were discharged with some clinical improvement (improved GCS and vital signs) but with apparent neurologic sequelae.

**Fig 3:**
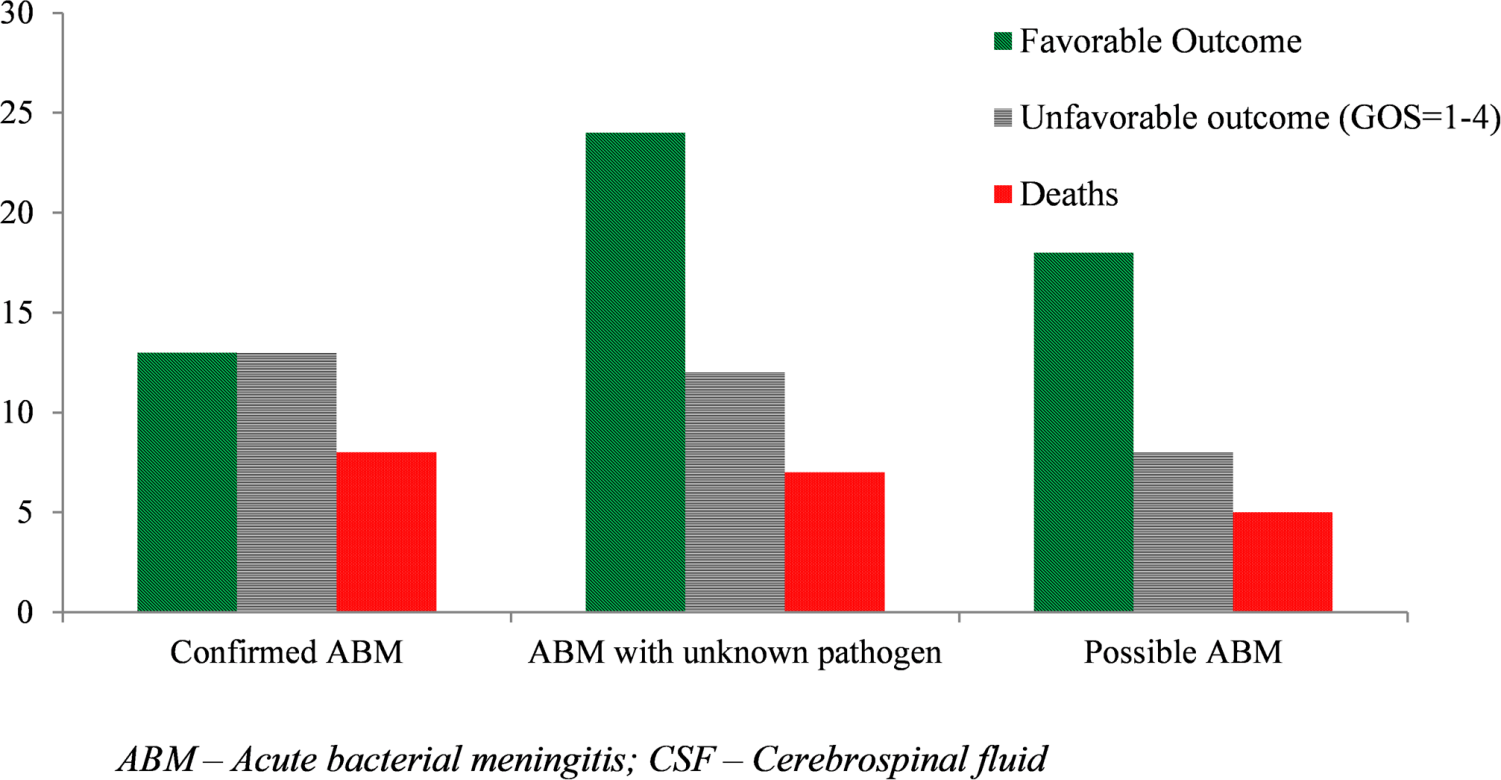
Discharge outcome by case category of patients treated as acute bacterial meningitis at Jimma University Hospital, Ethiopia.

Stratifying outcome according to case category, the rate of overall unfavorable outcome was found to be higher in those with proven ABM (50%) than probable cases (31.6%) and possible ABM cases (30.8%). The case fatality rate was also found to be higher in proven cases. However, the outcome differences between the groups are not statistically significant (Fig 3).

### Factors associated with poor outcome

A Bivariate analysis revealed that pulmonary crepitation, tachycardia, tachypnea, time to antibiotic treatment, low GCS on presentation, fever persisting despite two days of inpatient IV antibiotics, and treatment with adjunctive dexamethasone were found to be associated with overall unfavorable outcome (GOS<5). However, on multivariable analysis, low GCS (AOR = 0.766, 95% CI = 0.589–0.995), adjunctive dexamethasone therapy (AOR = 4.676, 95% CI = 1.12–19.50) and fever persisting after two days of antibiotics commencement (AOR = 24.226, 95% CI = 5.24–111.96) are factors independently associated with unfavorable outcome (Table 3).

**Table 3:** Factors associated with unfavorable outcome (GCS<5) in patients with acute bacterial meningitis at Jimma University Hospital, Ethiopia.

Variable (N = 90)	Outcome		Bivariate analysis		Multivariable analysis	
	Favorable (GCS = 5, N = 57)	Unfavorable (GCS = 1–4, N = 33)	COR (95% CI)	P	AOR (95% CI)	P
Age (years), Mean (SD)	33.11 (13.42)	32.21 (12.36)	0.999 (0.966–1.032)	0.946		
Sex, male	37 (64.9)	21 (63.64)	0.946 (0.387–2.312)	0.903		
Pre-hospital antibiotics	18 (31.58)	11 (33.33)	1.083 (0.434–2.703)	0.086		
HIV infected	8 (14.04)	8 (24.24)	1.960 (0.658–5.841)	0.227		
Pulmonary crepitation	7 (12.28)	12 (36.36)	4.082 (1.411–11.809)	0.009†	1.618 (0.260–10.073)	0.606
GCS, mean (SD)	13.72 (2.14)	11.45 (3.47)	0.743 (0.618–0.893)^a^	0.002†	0.766 (0.589–0.995)	0.046†
MAP, mean (SD)	85.26 (12.85)	82.53 (16.52)	0.986 (0.956–1.017)	0.381		
Pulse rate, mean (SD)	94.96 (13.40)	101.92 (20.74)	1.026 (0.999–1.054)	0.061		
Respiratory rate, mean (SD)	25.16 (5)	28.64 (8.23)	1.085 (1.013–1.162)^b^	0.02†	0.931 (0.819–1.058)	0.274
Seizure	13 (22.81)	7 (21.21)	0.911 (0.322–2.575)	0.861		
Focal neurologic deficit	2 (3.5)	1 (3.3)	0.859 (0.075–9.857)	0.903		
Time to antibiotics (hours), Mean (SD)	88.07 (68.36)	133.82 (98.39)	1.007 (1.001–1.012)^b^	0.016†	1.006 (0.998–1.014)	0.140
Adjunctive dexamethasone	12 (21.05)	18 (54.55)	4.500 (1.766–11.467)	0.002†	4.676 (1.12–19.50)	0.034†
CSF appearance (turbid)	31/32 (96.9)	21/22 (95.5)	2.117 (0.803–5.584)	0.130		
CSF WBC (cells/μL)^c^, median (IQR)	800 (2329)	630 (2201)	1.025 ((0.651–1.613)	0.915		
CSF protein (mg/dl),median (IQR)	102 (116)	13135 (136)	1.001 (0.997–1.006)	0.632		
Glucose ratio, mean (SD)	0.42 (0.26)	0.33 (0.11)	0.084 (0.004–1.625)	0.101		
Causative bacteria, *S*. *pneumoniae*	5/13 (38.5)	8/13 (61.5)	2.560 (0.527–12.431)	0.244		
Fever persisting after 48hr	6 (10.5)	22 (66.67)	31.17 (9.05–107.39)	<0.001†	24.226 (5.24–111.96)	<0.001†
Hemoglobin (g/dL), mean (SD)	12.31 (2.81)	12.25 (3.03)	0.993 (0.855–1.153)	0.928		

AOR—adjusted odds ratio; CI—confidence interval; COR—crude odds ratio; GCS—Glasgow coma scale; HIV—human immunodeficiency virus; MAP—mean arterial pressure

^**a**^ odds ratio decreases with unit increase in GCS

^b^ odds ratio increase for unit increase in predictor variable

^c^ odds ratio calculated on log_10_^CSF WBC^

^**†**^ Statistically significant

## Discussion

Our study has revealed that the diagnosis of bacterial meningitis was confirmed in only less than one third of the cases. It also showed that *Streptococcus pneumoniae* and *Neisseria meningitidis* ACYW were the most common etiologies of proven ABM at the hospital. Moreover, patients treated for suspected bacterial meningitis at the hospital suffered from a high rate of unfavorable outcome (36.7%) and high case fatality (22.2%). Depressed level of consciousness on presentation, treatment with adjunctive dexamethasone and fever persisting after two days of inpatient IV antibiotics treatment were found to be independently associated with unfavorable outcome.

Bacterial meningitis is known for its abrupt onset of symptoms. As a result, most patients present within the first two days of symptom onset [9, 14]. However, 4.5 days of symptoms on average before hospital presentation in our participants is longer than most reports from western literatures [9, 14, 38]. It is obviously difficult to compare findings in our study with as little as 29% confirmed cases with studies with established ABM cases; however, this reflects the complex situation in resource limited countries [13]. Nevertheless, the delay in hospital presentation may be explained by patient and healthcare related factors. Poor health seeking by patients, limited access to healthcare facility, misdiagnosis of ABM for simple febrile illness at primary care settings, and lack of proper referral system might have contributed to the delay. This was evidenced by the fact that 64.4% of the participants were rural residents and had to travel a median of 30 km (IQR = 50) to arrive at the hospital.

The diagnosis of ABM is confirmed based on microbiologic analysis of properly collected CSF specimen [1, 39]. However, in patients presenting after commencement of antibiotic treatment, the diagnosis of ABM is hampered by negative CSF culture and derangement of classic CSF profile for pyogenic meningitis. In our study, 32.2% presented after taking either oral or parenteral antibiotics in the community for similar complaints. Overall, 52.9% of patients whose CSF was analysed have already taken at least a dose of antibiotics before lumbar puncture. This might have resulted in low culture positivity rate and lower proportion of confirmed cases. Nevertheless, the fact that 84% of patients presented with classic triads of fever, headache and nuchal rigidity and that 65.9% had purulent CSF findings strongly support the possibility of pyogenic meningitis in many of those who did not have an identifiable pathogen.

Even though pre-emptive initiation of antibiotics in patients suspected to have ABM is essential to prevent dreadful outcomes [39–41], if no contraindications exist, CSF should be collected early, before antibiotic initiation. This does not only help to confirm the diagnosis but also to guide proper antibiotic choice for the treatment. This is particularly important in settings where data for etiologies and antibiotic susceptibility pattern is limited. Moreover, empiric antibiotic administration in such patients does not only hamper the diagnosis but also delays proper treatment and may consequently lead to poor outcome [11, 42].

Consistent with the findings for adult ABM [1, 14], most of the confirmed cases (84.6%) were due to either *Streptococcus pneumoniae* or *Neisseria meningitidis* ACYW. Due to the small number of confirmed cases, the figure may not reflect the real pattern of bacterial meningitis in the setting. However, the finding is in line with other data from Ethiopia [34, 43, 44]. Moreover, as diagnosis of tuberculous meningitis in Ethiopia, including this study, is hampered by limited diagnostic options. It is highly possible that more patients could have had TBM than already considered by treating physicians. Though there is no data for aseptic meningitis in the country, the possibility of viral meningitis/encephalitis particularly in those with HIV infection in such setting should also be considered.

Reasons for treatment delay may be complex and related to multiple factors such as poor health seeking behaviour, poor infrastructure and healthcare related factors. Nevertheless, its prognostic value is proven to be important [40, 41] and has been focus for guideline changes because of its amenability to interventions [45]. However, in settings with fragile healthcare system and where health seeking behaviour is low; referral linkage between primary care and tertiary centres is poor; and other infrastructures (roads and transportation) are underdeveloped, shortening time to treatment needs relentless effort from all stakeholders in the country.

Adjuvant dexamethasone in the treatment of ABM remains controversial despite multiple clinical trials. While studies from high income settings have consistently proven its survival benefit and improved overall outcome [46–49], most studies from low and middle income settings have shown none of such benefits [28, 38, 50, 51]. As a result, the use of adjunctive corticosteroid in such settings, particularly in those with high rate of HIV infection, has been discouraged. Despite these facts, the Ethiopian standard treatment guideline recommends the use of adjuvant dexamethasone in patients with suspected bacterial meningitis [25]. Consistently, 33.3% of patients in this study were given dexamethasone by their treating physicians. Its use was associated with unfavorable outcome (AOR = 4.676, 95% CI = 1.12–19.50). These findings are concordant with a recent retrospective study at four teaching hospitals in Ethiopia [36]. These data show that the use of dexamethasone as adjunctive treatment in ABM in low income settings does not confer any benefit and may be potentially harmful particularly in unconfirmed cases.

As detailed earlier, prompt initiation of antibiotics in patients suspected to have bacterial meningitis is essential to improve the overall outcome of patients. However, subsequent management of these patients should be guided by findings of CSF analysis and alternative diagnosis should be considered in the absence of supportive evidence for ABM. Most of these alternative diagnoses should also be managed promptly to improve the outcome. Therefore, careful clinical judgement is mandatory to keep the balance of proper pre-emptive management of ABM and avoiding misdiagnosis of other CNS conditions. A 2.4 days delay of treatment for alternative diagnosis was witnessed in this study. This delay in treatment in some of the differential diagnoses may lead to poor outcome. Of all these differential diagnosis, tuberculous meningitis deserves due attention due to its epidemiology and the availability of effective treatment.

The main strength of this study is that it has tried to assess outcome of patients admitted with diagnosis of ABM—reflecting the real life situation. However, our study is limited as it is a single centre study from a teaching hospital with a relatively better setting compared to the average situation encountered in Ethiopia. As a result, the reported outcome may not be representative for the whole nation. Secondly, the outcomes reported included only discharge conditions and overt neurologic sequelae. Thus, the real mortality and morbidity related to ABM in the setting may be more than stated here. Moreover, the small number of confirmed cases might have underpowered the effect of common outcome predictors. The lack of more sensitive diagnostics for TBM might have also resulted in its miss-diagnosis for ABM.

## Conclusion

A high mortality rate and unfavorable outcome was noted in patients treated as cases of bacterial meningitis at Jimma University Hospital in Ethiopia. Impaired level of consciousness on presentation, dexamethasone adjunctive therapy, and fever persisting two days after commencement of antibiotic treatment were found to be associated with overall poor outcome. Overall, assessment of treatment outcomes and risk factors was hampered by low rate of culture confirmed cases. Hence, locally applicable diagnostics and guidelines that enhance early and accurate diagnosis of patients with suspected meningitis is essential to improve patient outcome.

## Supporting information

S1 Dataset Acute bacterial meningitis in Ethiopia. (SAV)
